# Within‐household clustering of genetically related *Plasmodium falciparum* infections in a moderate transmission area of Uganda

**DOI:** 10.1186/s12936-021-03603-7

**Published:** 2021-02-02

**Authors:** Jessica Briggs, Alison Kuchta, Max Murphy, Sofonias Tessema, Emmanuel Arinaitwe, John Rek, Anna Chen, Joaniter I. Nankabirwa, Chris Drakeley, David Smith, Teun Bousema, Moses Kamya, Isabel Rodriguez-Barraquer, Sarah Staedke, Grant Dorsey, Philip J. Rosenthal, Bryan Greenhouse

**Affiliations:** 1 Department of Medicine, University of California San Francisco, San Francisco, CA USA; 2 Infectious Diseases Research Collaboration, Kampala, Uganda; 3 Department of Medicine, Makerere University College of Health Sciences, Kampala, Uganda; 4 Department of Immunology and Infection, London School of Hygiene and Tropical Medicine, London, UK; 5 Institute for Health Metrics & Evaluation, University of Washington, Seattle, WA USA; 6 Department of Medical Microbiology, Radboud University Nijmegen Medical Centre, Nijmegen, The Netherlands; 7 Department of Clinical Research, London School of Hygiene and Tropical Medicine, London, UK

**Keywords:** Malaria, Genotyping, Uganda, Transmission, Microsatellite, Clustering, Molecular epidemiology

## Abstract

**Background:**

Evaluation of genetic relatedness of malaria parasites is a useful tool for understanding transmission patterns, but patterns are not easily detectable in areas with moderate to high malaria transmission. To evaluate the feasibility of detecting genetic relatedness in a moderate malaria transmission setting, relatedness of *Plasmodium falciparum* infections was measured in cohort participants from randomly selected households in the Kihihi sub-county of Uganda (annual entomological inoculation rate of 27 infectious bites per person).

**Methods:**

All infections detected via microscopy or *Plasmodium-*specific loop mediated isothermal amplification from passive and active case detection during August 2011-March 2012 were genotyped at 26 microsatellite loci, providing data for 349 samples from 230 participants living in 80 households. Pairwise genetic relatedness was calculated using identity by state (IBS).

**Results:**

As expected, genetic diversity was high (mean heterozygosity [H_e_] = 0.73), and the majority (76.5 %) of samples were polyclonal. Despite the high genetic diversity, fine-scale population structure was detectable, with significant spatiotemporal clustering of highly related infections. Although the difference in malaria incidence between households at higher (mean 1127 metres) *versus* lower elevation (mean 1015 metres) was modest (1.4 malaria cases per person-year *vs.* 1.9 per person-year, respectively), there was a significant difference in multiplicity of infection (2.2 *vs.* 2.6, p = 0.008) and, more strikingly, a higher proportion of highly related infections within households (6.3 % vs. 0.9 %, p = 0.0005) at higher elevation compared to lower elevation.

**Conclusions:**

Genetic data from a relatively small number of diverse, multiallelic loci reflected fine scale patterns of malaria transmission. Given the increasing interest in applying genetic data to augment malaria surveillance, this study provides evidence that genetic data can be used to inform transmission patterns at local spatial scales even in moderate transmission areas.

## Background

Falciparum malaria remains one of the most important infectious diseases in the world, causing significant morbidity and mortality. More than 90 % of malaria deaths occur in sub-Saharan Africa, with the majority of these in children less than 5 years old [1]. Considerable progress has been made in the past decade in reducing the burden of malaria in Africa, largely due to interventions such as long-lasting insecticidal nets (LLINs), indoor residual spraying (IRS), and the use of artemisinin-based combination therapy [2]. However, the effectiveness of such large-scale interventions is non-uniform in part due to significant heterogeneity in baseline malaria transmission, demonstrating the need for targeted control and elimination programs that prioritize the most successful techniques [3]. To most efficiently target interventions, the ability to stratify risk within a population is necessary, potentially including an understanding of fine-scale transmission. Traditional methods of evaluating transmission lack the resolution to differentiate the burden of disease over small spatial scales, especially in high transmission areas like Uganda, where an estimated 12 million clinical cases are treated annually in the public health system alone [4].

As with most infectious diseases, malaria transmission within a geographical area is heterogeneous and can vary greatly between villages and between households in a village [5–10]. Variations in malaria burden may reflect differences in vector distribution by habitat, human-vector contact, and human host factors [5, 8]. More specifically, spatial clustering of malaria cases is well-described, which can be observed at scales as small as the household level [8, 9]. While it has been proposed that parasite genetic data can be used to identify active clusters of infection, or “hotspots”, and to assess their contribution to onward transmission, these techniques are best established in areas of relatively low transmission, and it is less clear if these techniques can identify clustering of related infections in moderate to high transmission settings. To evaluate whether parasite genetic data can be used to elucidate heterogeneity in malaria transmission at fine spatial scales in a moderate transmission area, this study analysed data from 26 microsatellites in 349 *Plasmodium falciparum* infections identified in participants living in 80 households in Kihihi, Uganda.

## Methods

### Study design and participants

Samples for this study were obtained from a previously described cohort study conducted in Kihihi sub-county, Kanungu District in southwestern Uganda, an area spanning approximately 12 × 24 km^2^ in southwestern Uganda [11–13]. Kihihi sub-county is predominantly rural and has a moderate malaria transmission intensity, with an estimated annual entomological inoculation rate (EIR) of 27 in 2012 [13]. Briefly, participants were recruited from 80 randomly selected households within the catchment area of the participating health facility. All children aged 6 months to 10 years and a primary adult caretaker (at least 18 years of age) in a household were enrolled. Participants agreed to come to the study clinic for any febrile illness and to avoid anti-malarial medications administered outside the study. Study participants attended the clinic at enrollment and then every 90 days for routine visits. Participants who reported a history of fever in the previous 24 hours or had a tympanic temperature ≥ 38.0 °C and had a positive blood smear were diagnosed with malaria and treated with artemether-lumefantrine. At routine visits, participants received a history and physical examination and blood was obtained for thick blood smear and dried blood spots (DBS). DBS specimens were prepared by spotting whole blood onto filter paper and drying completely. If a participant was microscopy negative at a routine visit, loop-mediated isothermal amplification (LAMP) was performed to assess for submicroscopic parasitaemia.

**Fig. 1:**
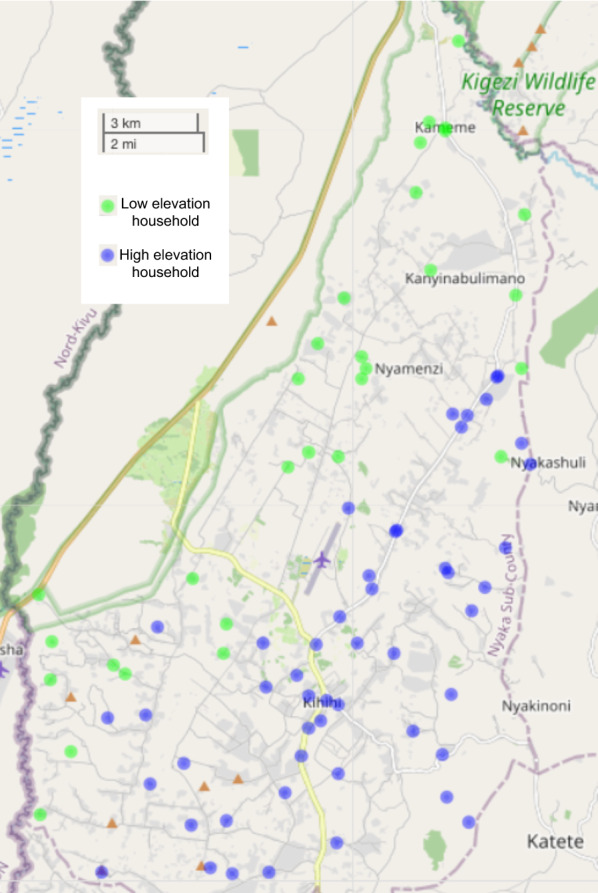
Map of study area showing cohort households from which samples were collected. Blue households are at higher elevation (> 1075m) and green households are at lower elevation ( < = 1075m)

For this analysis, DBS samples from cohort participants who were parasitaemic by microscopy or LAMP from August 2011 to March 2012 were included. In addition, DBS samples were included from additional household adults (not enrolled in the cohort) who provided a one-time dried blood spot sample within 30 days of household enrollment for genotyping. Median elevation of households from which samples were collected was 1075 m (min 949 – max 1311 m). For simplicity, participants living in households above the median elevation were defined for the purposes of this study as living at higher elevation, while those at or below the median were considered to be at lower elevation (Fig. 1). Entomological surveys, in which mosquitoes were collected once a month from each household using miniature CDC light traps, as described previously, were also performed [12]. Malaria incidence and entomological data are reported for all households in the cohort for the first full year of the study (August 2011–August 2012).

### Laboratory methods

To determine presence of parasitaemia, samples were evaluated by microscopy and *Plasmodium*-specific LAMP as previously described [14]. Briefly, thick blood smears were prepared with 2 % Giemsa. Light microscopy was performed by an experienced laboratory technician who was not involved in direct patient care. A second technician performed quality control for detection of parasites for all microscopy results and a third technician resolved discrepancies if needed. LAMP was performed on all DBS specimens for which a participant had a negative blood smear. DNA was extracted for LAMP using saponin-Chelex extraction, as previously described [14]. LAMP was performed using Eiken Loopamp™ Malaria Pan Detection Kit reaction tubes [15]. For all samples positive by light microscopy or LAMP, parasite density was quantified using varATS qPCR as described previously [16]. Samples with parasite density > 5 parasites/µL of blood were then genotyped using 26 microsatellite loci [17]. Two rounds of PCR were conducted for all loci, and PCR products were diluted and sized by capillary electrophoresis using an Applied Biosystems 3730xl DNA Analyzer. Alleles were sized from electropherograms using microSPAT software, which includes a probabilistic algorithm to filter artifacts [18].

### Data analysis

**Fig. 2:**
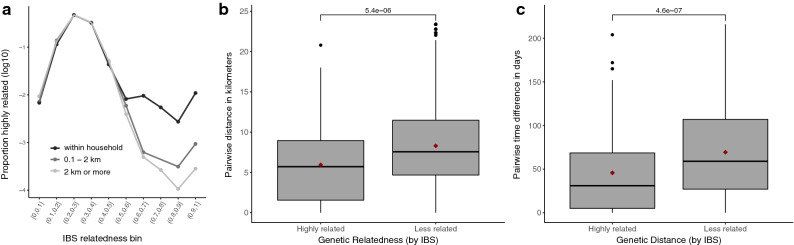
**a** Highly related pairs of infections (Identity by State (IBS) > = 0.6) contain the majority of spatially informative genetic signal. **b** Highly related infections were closer spatially (mean = 5.9 km) than less related infections (mean = 8.3 km). **c** Highly related infections were also more likely to occur within a shorter timespan (mean 45.7 days vs. 69.4 days). Median and IQR represented by boxplot; mean indicated by red point. P-values computed for comparison of means computed using Wilcoxon test

All data were analysed using R (version 3.5.0) with R studio (version 1.1.453; Rstudio team). Expected heterozygosity (H_e_) was calculated by using the formula H_e_ = [n/(n – 1)] [(1 – ∑P_i_^2^ )], where n = sample size and P_i_ = allele frequency. The mean multiplicity of infection (MOI) was calculated by dividing the total number of clones by the number of PCR-positive samples for each marker gene. Pairwise time was calculated as the difference in days between obtaining the two samples. Pairwise spatial distance was calculated as the Euclidian distance in metres between the households from which samples were obtained. Pairwise identity by state (IBS) was calculated from allele similarity between isolates using a modified IBS metric to measure parasite relatedness [19–21]. Briefly, IBS was computed based on the number of shared alleles between pairs of infections in both monoclonal and polyclonal infections. The overall pairwise IBS was calculated as $$\frac{1}{n} {\sum }_{i=1}^{n}\begin{array}{c}\frac{{S}_{i}}{{X}_{i}{Y}_{i}}\end{array}$$, where *n* = number of genotyped loci, S_*i*_ = total number of shared alleles at locus *i* between samples X and Y; X_*i*_ = number of alleles in sample X at locus *i* and Y_*i*_ = number of alleles in sample Y at locus *i*. An IBS of ≥ 0.6 was used as a cut off for the most highly related samples, as per Fig. 2a. This is similar to other studies in which a cut off of ≥ 0.5 was used [20, 21].

Negative binomial regression was used to estimate malaria incidence and to calculate incident rate ratios (IRR). Comparisons of proportions were performed using generalized estimating equations to account for repeated measures. Pairwise distance and time means were compared using the Wilcoxon test for independent samples.

## Results

### Transmission intensity by elevation

Despite the small size of the study area, there were noticeable differences in transmission intensity comparing cohort households above the median elevation (higher elevation) *versus* below the median (lower elevation). Using data from all children in the cohort through one year, there was lower malaria incidence in children living at higher compared to lower elevation (1.4 versus 1.9 episodes per person-year (ppy) (IRR = 0.73, 95 % CI: 0.53–1.00). In addition, the daily human biting rate for anopheline mosquitoes (dHBR) was considerably lower in households at higher versus lower elevations (2.01 vs. 8.9, p = 0.08), adding evidence to support higher transmission at lower elevation. Thus, the data suggest modest but meaningful differences in transmission within the study area by both clinical and entomologic measures, with transmission higher in houses at lower elevation.

### Study population and genotyping

A total of 408 samples with ≥ 5 parasites/µL of blood from 80 households were genotyped using 26 microsatellite markers. Of these, 349 (85.5 %) samples were successfully genotyped at 15 or more loci and included in subsequent analysis; these samples were collected from 230 unique individuals (142 children and 88 adults) (Table 1). Of these, 128 (51.2 %) children and 4 (4.0 %) adults reported and/or had objective fever at the time of their sampled infections.

### Complexity and genetic diversity of infections

The majority of genotyped infections were polyclonal (76.5 %) with a mean MOI of 2.4 (range = 1–6, Additional file 1: Fig. S1 and Table S1). Children tended to have less complex infections, with 30.0 % monoclonal infections in children compared to only 7.1 % monoclonal infections in adults (p = 0.0002, Table 1). Samples from lower elevation households had a higher mean MOI than samples from the higher elevation households (2.6 vs. 2.2, p = 0.008); this was driven by differences in MOI between children at lower vs. higher elevation (2.4 vs. 2.1, p = 0.024). However, populations at both elevations had a similar proportion of monoclonal infections: 24.9 % for higher elevation and 22.2 % for lower elevation samples (p = 0.56). The overall population level genetic diversity was high in Kihihi, with a mean heterozygosity (H_e_) of 0.73 (range: 0.37–0.91) with no difference between elevations or between adults and children.

**Table 1:** Descriptive statistics of 349 successfully genotyped *P. falciparum* infections from the Kihihi Subcounty of Uganda

	Children (0.5–11 years)	Adults (> 18 years)	Total
Number of individuals	142	88	230
Samples (coverage at > = 15 markers)	250	99	349
Symptomatic infections, n (%)	128 (51.2 %)	4 (4.0 %)	132 (37.8 %)
Microscopy positive, n (%)	161 (64.4 %)	5 (5.1 %)	166 (47.6 %)
Monoclonal infections, n (%)	75 (30.0 %)	9 (7.1 %)	82 (23.5 %)

	Higher Elevation	Lower Elevation	Total
Unique Households	50	30	80
Avg number of children/household (mean)	1.6	2.1	1.8
Average child age in years (mean)	5.8	5.5	5.6
Monoclonal infections, n (%)	43 (24.9 %)	39 (22.2 %)	82 (23.5 %)
Mean Heterozygosity	0.72	0.72	0.73
Mean Multiplicity of Infection	2.2	2.6	2.4

### Spatial scale of genetic relatedness

Pairwise genetic relatedness was determined between all genotyped samples obtained from different individuals, including those with polyclonal infections, resulting in 60,568 pairwise comparisons. Of these, there were 733 intra-household pairs and 3,306 pairs of monoclonal infections. Infections from the same household were more likely to be genetically related than infections from participants that did not (Fig. 2a); a similar result was seen when limiting this analysis to only monoclonal samples. There was suggestion of a small increase in relatedness between infections from participants in different households living < 2 km vs. >= 2km apart, though statistical power to evaluate this question was limited due to small numbers. A total of 93 (0.14 %) infection pairs were highly related (IBS > = 0.6). Highly related pairs showed significant spatiotemporal clustering compared to those with IBS < 0.6. (Fig. 2b, c). 79/93 (84.9 %) of highly related infections were from participants who lived within 10km of each other, and 78/93 (83.9 %) of highly related infections occurred within 90 days of each other.

### Within‐population and within‐household transmission

**Fig. 3:**
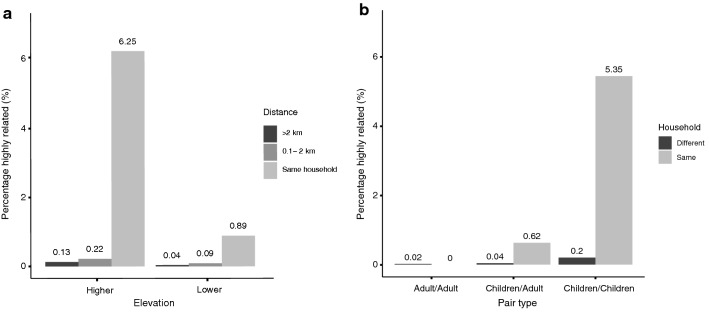
**a** Percentage of highly related infections by distance, stratified by elevation. **b** Percentage of highly related infections in children-children, children-adult, or adult-adult pairs between and within households

On a finer spatial scale, a higher proportion of highly related infections was found in members of the same household compared to individuals living in different households (2.9 % vs. 0.1 %, p < 0.0001), suggesting that transmission-related infections cluster within households. To test the hypothesis that highly related infections were more likely to be observed within the same household where transmission was lower, the proportion of highly related infections within households was compared by elevation. As hypothesized, a higher proportion of highly related pairs of infections was found within households at higher elevation compared to lower elevation (6.3 % vs. 0.9 %, p = 0.0005) (Fig. 3a).

There were too few within-household highly related infection pairs (n = 21) to evaluate within-household transmission events. However, the proportions of highly related infections in child-child, child-adult, and adult-adult paired samples both within and between households were calculated. In child-child pairwise comparisons, the proportion of highly related infections was higher than in comparisons for child-adult or adult-adult pairs (Fig. 3), although the greater MOI observed in adults complicated interpretation of this result. However, when the analysis was restricted to monoclonal samples, there was again a higher proportion of highly related samples in child-child pairs than in child-adult pairs (0.5 % vs. 0.06 %, p = 0.005), and no adult-adult pairs were highly related. As might be expected given other evidence for within-household clustering, this pattern was more pronounced within households, with 5.4 % of child-child pairs within households highly related compared to 0.2 % of child-child pairs from different households (p < 0.0001) (Fig. 3). When the analysis was restricted to monoclonal samples, this pattern held, with 6.8 % of child-child pairs within-households highly related compared to 0.4 % of child-child pairs from different households (p < 0.0001).

## Discussion

To evaluate the potential to detect genetic relatedness of malaria parasites in a moderate malaria transmission setting, relatedness of infections was measured using 26 microsatellites in Kihihi sub-county, southwestern Uganda. As expected, genetic diversity was high and the majority of samples were polyclonal. Despite the high diversity, spatiotemporal structure in genotypes was detectable, with significant spatial and temporal clustering of highly related infections identified in both higher and lower transmission areas of Kihihi, including marked clustering of related infections within households. Despite modest differences in malaria incidence found at higher vs. lower elevations within the study site, there were differences in MOI and spatial clustering of infections, reflecting underlying differences in transmission identified using detailed entomologic measures. Of note, statistical power for both of these molecular metrics was higher than that observed for malaria incidence. Thus, these data demonstrate the potential utility for genetic measures of parasite diversity in stratifying malaria transmission, even in moderate to high transmission settings where the majority of infections are polyclonal.

While it is clear that there is heterogeneity in malaria transmission at all levels of endemicity, this phenomenon is most obvious in areas of low transmission [8, 22]. Therefore, genetic epidemiology of malaria often focuses on low transmission settings. For example, to look at the contribution of importation *versus* local transmission in Namibia, Tessema et al.. showed that microsatellite data could capture strong spatial signals over both local and regional scales in a study with limited travel history and without cross-border mobile phone data. In a study conducted along the Thai-Myanmar border, single nucleotide polymorphism (SNP) barcode data were used to show that identity by descent (IBD), a measure of parasite relatedness, declined with increasing inter-clinic distance [23]. In Zambia, a country targeted for elimination, Pringle et al. used microsatellite genotyping of samples collected through reactive case detection (RCD) to show that participants from the same RCD event harboured more genetically related parasites than those from different RCD events [20]. Two other studies in moderate to high transmission areas showed clustering of infections at distances of < 1km [24] or at the household level [25], using SNP genotyping and amplicon sequencing, respectively. Other molecular epidemiology studies of malaria in settings of various endemicity have failed to observe any parasite population genetic structure, either geographically or temporally [26–28]. Notably, using only 349 samples from 80 households, within-household clustering of infections was detected, suggesting that genetic data in this setting reflect what is known about clustering of malaria at the household level from epidemiological studies [22]. These data show that by focusing on the most highly related infections in a population, as prior studies have done in lower transmission settings, micro-epidemiological patterns can be revealed even in a moderate transmission setting.

Additionally, this study showed that relatedness of infections between children was greater than that of infections between children and adults or infections between adults. This pattern was evident both between and within households, but was much more pronounced within households. This pattern may be due to age-related immunity, since adults may more readily suppress or clear a proportion of parasites to which they had been previously exposed. Alternatively, because children harbour higher parasite densities than adults, they may be more likely to transmit to other children, including those in the same household [29]. A recent study by Nelson et al. that employed targeted amplicon deep sequencing to genotype parasites found that symptomatic children more commonly shared haplotypes with asymptomatically infected household members; however, household members were not stratified by age in this study [25]. Understanding fine-scale transmission networks and whether household clustering is driven by transmission between household members or whether household members acquire related infections from exposure to the same vector would be helpful in determining the best method for spatially targeting malaria interventions in lower transmission settings, including interventions targeting the human parasite reservoir.

The study had some limitations. For example, IBS is a biased measure of parasite relatedness if one is attempting to compare IBS measures across different studies or populations, because it is sensitive to allele frequencies [30]. An advantage of this measure, however, is its simplicity and applicability to polyclonal samples, and it has been used successfully in other studies [20, 21]. Ideally, computationally tractable but more generalizable metrics of relatedness applicable to multiallelic, polyclonal samples would provide more robust analyses from this type of data. Unfortunately, methods to calculate such metrics, e.g. unbiased estimates of identity by descent (IBD) from these types of data are not currently available. Another limitation is the small sample size, with only 349 genotyped infections, which did not allow analyses to determine the dynamics of within-household transmission. However, this sample size was sufficient to observe clustering of highly related infections and differentiate neighbouring geographic regions that had only modest differences in transmission intensity. Notably, the ability to detect signal in small sample sizes is an advantage in studies that aim to utilize genetic data for surveillance due to lower cost and easier collection. Another strength of this study was the ability to capture information from polyclonal infections, leveraging the high diversity and multiallelic nature of the microsatellite loci. Each time an analysis in this study was replicated using only monoclonal samples, a similar but less well-defined pattern was found, suggesting that polyclonal samples contribute important information and help to better define aspects of population structure relevant for transmission. However, while the multiallelic, high-diversity nature of microsatellite loci has some advantages, this type of marker can be cumbersome to evaluate, particularly when considering larger numbers of loci to more accurately define relatedness between infections. Other genotyping techniques that are able to resolve multiallelic loci, such as multiplex amplicon or “microhaplotype” sequencing, can provide rich data from polyclonal infections and leverage current sequencing technologies to more easily allow evaluation of larger numbers of loci, providing higher resolution to evaluate transmission particularly in moderate-high transmission settings [25, 31–34]. While these methods are more expensive and require more extensive laboratory and bioinformatics structure than the methods used in this study, they may be preferred techniques in the future as sequencing costs continue to decline.

## Conclusions

In this study, spatiotemporal clustering of malaria infections was identified in a moderate transmission setting by analysing all infections and focusing on those which were highly related. There was also evidence of clustering of related malaria infections within households, which was more evident in areas of lower transmission. Importantly, genetic data were able to clearly discriminate transmission intensity in this small study area where only modest differences in malaria incidence were evident. Given the increasing interest in applying genetic data to augment malaria surveillance, this study provides evidence that genetic data can be used to inform transmission patterns at local spatial scales even in moderate transmission areas.

## Supplementary Information

**Additional file 1: Table S1.** Diversity at each microsatellite marker. Avg He 0.73.** Figure S1.** MOI distribution. A) Overall distribution of MOI B) Distribution of MOI at lower elevation C) Distribution of MOI at higher elevation.

## Data Availability

The full PRISM epidemiological dataset is available at ClinEpiDb.org at: https://clinepidb.org/ce/app/record/dataset/DS_0ad509829e. The microsatellite data used in this study can be found at: https://github.com/EPPIcenter/Kihihi_MS_genotyping.
