# Supplementary material for: Within‐household clustering of genetically related Plasmodium falciparum infections in a moderate transmission area of Uganda
Source: Malar J. 2021 Feb 2;20:68. doi: 10.1186/s12936-021-03603-7 (PMC8042884; doi:10.1186/s12936-021-03603-7)
Supplement: Supplementary file 1 — Additional file 1: Table S1. Diversity at each microsatellite marker. Avg He 0.73. Figure S1. MOI distribution. A) Overall distribution of MOI B) Distribution of MOI at lower elevation C) Distribution of MOI at higher elevation. [file 12936_2021_3603_MOESM1_ESM.docx]

**Additional Material**

| **Loci** | **Total Number of Samples** | **He** | **Frequency of unique alleles** |
| --- | --- | --- | --- |
| AS1 | 379 | 0.373236 | 6 |
| AS2 | 427 | 0.781991 | 9 |
| AS3 | 478 | 0.862258 | 11 |
| AS32 | 422 | 0.670702 | 14 |
| AS7 | 341 | 0.407152 | 4 |
| AS8 | 375 | 0.572796 | 6 |
| AS11 | 467 | 0.696728 | 9 |
| AS12 | 431 | 0.576449 | 5 |
| AS14 | 412 | 0.761331 | 13 |
| AS15 | 487 | 0.838911 | 13 |
| AS19 | 304 | 0.624587 | 7 |
| AS21 | 309 | 0.520069 | 5 |
| AS34 | 410 | 0.513373 | 6 |
| AS25 | 558 | 0.87599 | 23 |
| B7M19 | 423 | 0.609322 | 10 |
| TA109 | 592 | 0.838387 | 20 |
| AS31 | 479 | 0.77306 | 17 |
| Ara2 | 478 | 0.825871 | 11 |
| PfPK2 | 547 | 0.867574 | 21 |
| TA1 | 571 | 0.90873 | 23 |
| TA87 | 644 | 0.901268 | 17 |
| TA81 | 570 | 0.82103 | 13 |
| TA60 | 453 | 0.833851 | 11 |
| PolyA | 740 | 0.94846 | 34 |
| PFG377 | 446 | 0.616685 | 9 |
| TA40 | 534 | 0.900672 | 21 |

**Additional Table S1:** Diversity at each microsatellite marker. Avg He 0.73.


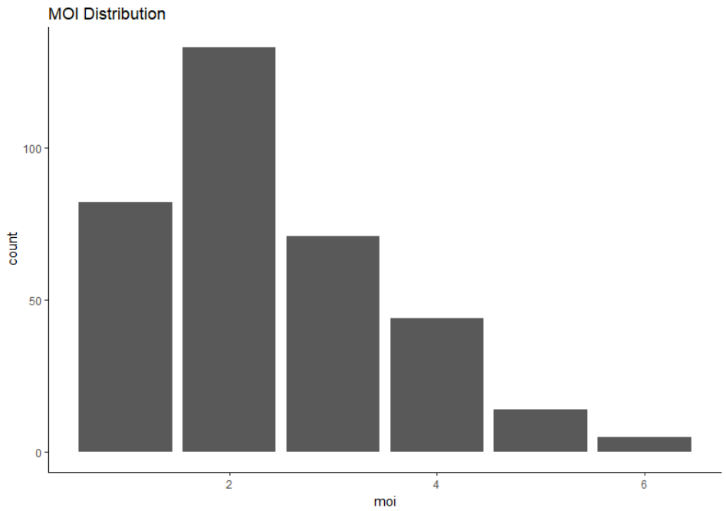

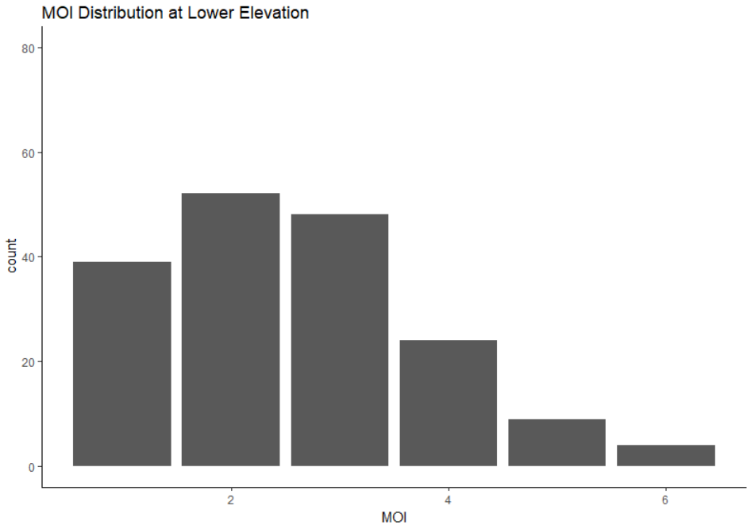

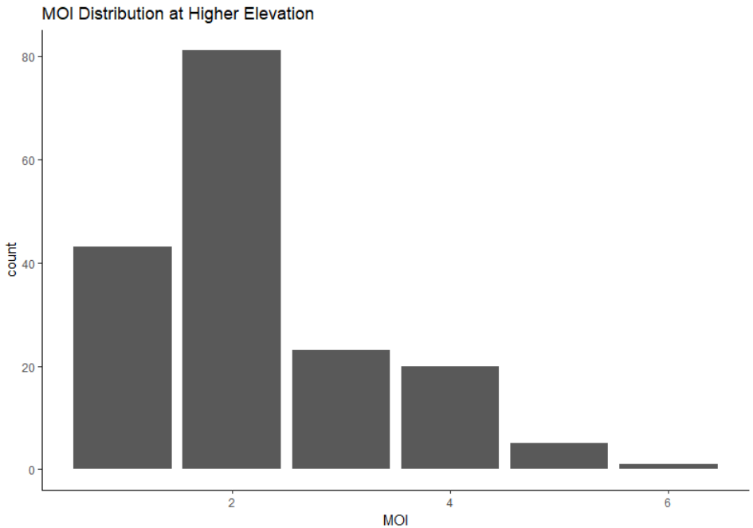


**Additional Figure S1:** MOI distribution. A) Overall distribution of MOI B) Distribution of MOI at lower elevation C) Distribution of MOI at higher elevation
